# Stickler Syndrome: A Review of Clinical Manifestations and the Genetics Evaluation

**DOI:** 10.3390/jpm10030105

**Published:** 2020-08-27

**Authors:** Megan Boothe, Robert Morris, Nathaniel Robin

**Affiliations:** 1 Department of Genetics, University of Alabama at Birmingham, Birmingham, AL 35233, USA; meganehlinger@uabmc.edu; 2 Retina Specialists of Alabama, Birmingham, AL 35233, USA; rmorris@rmeyes.com

**Keywords:** Stickler Syndrome, genetic testing, *COL2A1*, *COL11A1*, next-generation sequencing

## Abstract

Stickler Syndrome (SS) is a multisystem collagenopathy frequently encountered by ophthalmologists due to the high rate of ocular complications. Affected individuals are at significantly increased risk for retinal detachment and blindness, and early detection and diagnosis are critical in improving visual outcomes for these patients. Systemic findings are also common, with craniofacial, skeletal, and auditory systems often involved. SS is genotypically and phenotypically heterogenous, which can make recognizing and correctly diagnosing individuals difficult. Molecular genetic testing should be considered in all individuals with suspected SS, as diagnosis not only assists in treatment and management of the patient but may also help identify other at-risk family members. Here we review common clinical manifestation of SS and genetic tests frequently ordered as part of the SS evaluation.

## 1. Introduction

Stickler Syndrome (SS) is a relatively common multisystem connective tissue disorder. First described in 1965 by Gunnar Stickler [1], SS is best known to ophthalmologists as a condition that confers a risk for significant ocular complications, ranging from severe myopia to retinal detachment and vision loss [2,3,4]. While ocular complications are both common and often very serious, skeletal/joint, inner ear, and craniofacial structures are often involved [2,5]. Therefore, it should not be surprising that SS may present with a wide range of findings, including micrognathia (alone or as part of Pierre Robin sequence), cleft palate, hearing loss, or early onset osteoarthritis, either in the proband or an affected family member [5,6].

SS is also genetically complex. While the most common forms are autosomal dominant (AD), other forms are inherited in an autosomal recessive (AR) manner [2,6]. Furthermore, SS exhibits genetic heterogeneity, as to date, pathogenic variants in 6 different genes can cause SS [2,7,8,9,10,11]. These genes are associated with the formation of collagens type II, IX, and XI, which are expressed within the vitreous, skeleton, and inner ear, with limited genotype-phenotype correlation [2,3,9,10,11,12,13,14,15]. SS exhibits significant inter- and intrafamilial phenotypic variability, meaning that individuals with the same pathogenic variant may exhibit different clinical manifestations, even within families [6]. However, penetrance does appear to be complete, meaning all affected individuals will manifest some features of the condition [5,6]. A high index of suspicion allows for early detection and diagnosis which can improve clinical outcomes for these individuals [4,16,17].

Stickler Syndrome type I (STL1) is the most common form of SS, accounting for approximately 80–90% of cases [2,15]. STL1 is the classic AD type, caused by heterozygous pathogenic variants in *COL2A1* [2,5,15]. While most individuals with pathogenic *COL2A1* variants will exhibit systemic manifestations of SS, individuals harboring pathogenic variants in exon 2 will exhibit an ocular-only phenotype due to alternative splicing of the gene [18,19]. Ocular-only phenotypes may also be caused by variants in *COL2A1* outside of exon 2 [20,21,22]. Thus, a diagnosis of SS should be considered in an individual with suggestive ocular findings even if no systemic features are present [18,19]. A smaller proportion of AD cases are caused by heterozygous pathogenic variants in *COL11A1* (Stickler Syndrome Type II [STL2]), and *COL11A2* (Otospondylomegaepiphseal dysplasia, AD [OSMEDA], formerly known as Non-Ocular Stickler Syndrome) [7,8,13,14]. This is an important distinction for ophthalmologists, as individuals with OSMEDA will manifest all of the findings of typical SS except for the ocular complications [14]. This is because, while *COL11A2* is expressed in the joints, inner ear, and craniofacial structures, it is not expressed in the eye [14]. More rare than the AD forms, biallelic pathogenic variants in *COL9A1*, *COL9A2*, and *COL9A3* have been reported to cause recessive forms of Stickler Syndrome (Stickler Syndromes 4-6 [STL4-6]) [9,10,11,23]. Biallelic variant in *COL11A1* have also been reported in cases of AR SS, although typically are associated with a more severe skeletal dysplasia [24]. Biallelic variants in candidate genes, including *LOXL3*, a lysly oxidase gene important for proper cross-linking of type II collagen, and *LRP2*, an endoplasmic transmembrane receptor gene associated with facio-oculo-acoustico-renal (FOAR) syndrome and Donnai–Barrow Syndrome (DBS), have also been reported in individuals with SS-like phenotypes [25,26,27]. A family history is often absent in recessive cases, and clinical phenotypes may be less distinctive than those with STL1 and STL2 [9,10,11,23].

## 2. Clinical Characteristics

As discussed above, the clinical findings associated with SS may vary widely, even among affected members of the same family. However, as would be expected for a common condition that has been recognized for many years, the various complications have been well-described.

### 2.1. Ocular Manifestations

Individuals with SS are commonly myopic (>−3.00 diopters) and are at high risk for ocular complications, including vitreous abnormalities, retinal detachment, glaucoma, and cataracts [2,3,5,28,29,30,31]. High myopia is typically congenital and may be associated with astigmatism [2,3]. Congenital cataracts may also be present [2,3,32]. Some individuals exhibit congenital abnormalities of the anterior chamber drainage angle, increasing the risk for glaucoma [3,33]. Vitreous abnormalities are common with a high risk of retinal detachment [2,3,4,5,16,17,28]. Membranous vitreous is characteristically seen in STL1 [2,28,29]. Approximately 60–70% of individuals with STL1 will experience a retinal detachment, and of those approximately half will be bilateral [3,4,16,17]. Individuals with STL2 typically exhibit a beaded vitreous, although membranous vitreous has also been reported in these individuals [3,4,22,28]. Risk of retinal detachment in individuals with STL2 has been less widely studied than in those with STL1 but has been previously reported to be approximately 40% [28]. To date, high myopia, hypoplastic vitreous, and retinal detachment have been reported in STL4–6, although individuals with STL6 have also been reported without vitreous abnormalities [9,10,11,23,34]. The average age of retinal detachment in SS is between the ages of 10–30 years and is a significant cause of retinal blindness in children [4,16]. Those with retinal detachment often require multiple surgical interventions, with a high rate of recurrence and overall poor visual prognosis [4,16,17]. For this reason, prophylactic intervention has been explored to aid in vision preservation for these individuals and early detection and diagnosis is critical [16,17].

### 2.2. Craniofacial Manifestations

**Figure 1:**
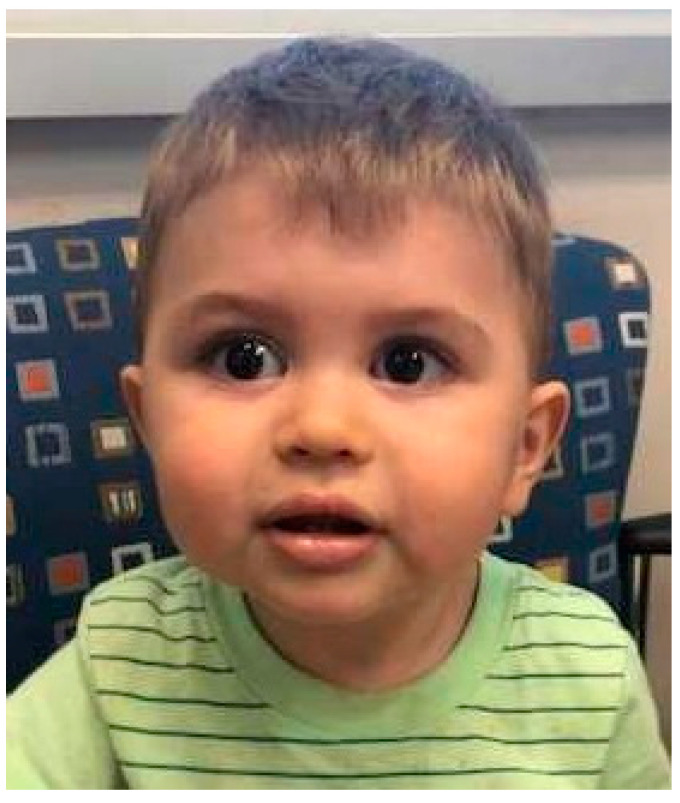
A child with classic STL1 due to a pathogenic variant in COL2A1 (c.28977_2878insTT). Characteristic midface hypoplasia and micrognathia are present.

Individuals with systemic forms of SS typically have underdevelopment of the maxilla, which leads to the appearance of a flattened facial profile known as midface hypoplasia (Figure 1) [2,5,28]. This is more apparent in childhood and may normalize in adulthood [6]. Micrognathia and Pierre Robin Sequence are common features, and clefts of the hard or soft palate may be present [2,5,6,28,31]. Cleft palate has not been observed in individuals with STL4–6, although variable degrees of midface hypoplasia and micrognathia have been described. It should be noted that clefting involving the lip occurs by a different embryologic mechanism and is not a feature of SS [2,5,28,35].

### 2.3. Auditory Manifestations

Hearing loss is a common feature of SS [2,5,28,31,36,37]. The most common form of hearing loss is pure sensorineural hearing loss, with increasing prevalence with age [36,38]. Mixed sensorineural and conductive hearing loss and purely conductive hearing loss may also be seen, more commonly affecting children or those with history of palatal defects [5,36]. Individuals with STL1 generally exhibit a mild to moderate sensorineural hearing loss that affects the higher frequencies [36,37,38]. In contrast, individuals with STL2 tend to have more pronounced hearing loss that is more apparent at younger ages [36,37,38]. Individuals with recessive forms of SS have been reported to have early onset sensorineural hearing loss of higher frequencies, with mild to moderate hearing loss described in STL5 and moderate to severe hearing loss in STL4 and STL6 [9,10,11,23,34].

### 2.4. Skeletal Manifestations

Skeletal features are common in individuals with systemic forms of SS [2,5,28]. Precocious arthritis is common, with symptoms ranging from mild to severe [2,5,6,39]. Spinal abnormalities, including scoliosis, kyphosis, and platyspondyly may also be seen, with a significant proportion of adults reporting chronic back pain [5,39,40]. Joint hypermobility may be present in childhood but typically resolves in adulthood [2,5,39].

## 3. Diagnosing Stickler Syndrome

Clinical diagnostic criteria have been proposed for STL1 but have not been validated [6,39]. These are based on a scoring system that assigns points to clinical features, family history, and molecular data, with “major” criteria receiving 2 points and “minor” criteria receiving 1 point [39]. A clinical diagnosis may be made if ≥5 criteria are met, at least one major feature is present, and there are no features suggestive of an alternative diagnosis [39].

These clinical diagnostic criteria may miss individuals with ocular-only and non-ocular phenotypes, as well as those with AR forms of SS or de novo AD cases in which a family history is absent. Thus, SS should be still be suspected in those with suggestive findings who do not meet clinical criteria.

Molecular genetic testing should be pursued in any individual for whom SS is suspected. In addition to confirming a diagnosis in the individual, a molecular genetic diagnosis can assist in the testing of at-risk family members, in guiding medical management and screening and in providing accurate recurrence risk for offspring [41]. A number of genetic tests may be considered in the genetic evaluation of suspected SS.

### 3.1. Chromosomal Microarray

Chromosomal microarray (CMA) is recommended as the first-tier testing in individuals with multiple congenital anomalies (MCA) or nonspecific developmental delays (DD) without features suggestive of a specific genetic disorder [42]. For that reason, many individuals with suspected SS will have a CMA. However, as this technology is designed to detect relative large missing or extra DNA segments (“copy number variations”), it is unlikely to be abnormal in a patient with SS [43]. If CMA does identify an abnormality, it is likely that the patient does not have SS but rather a condition that closely mimics it. However, because it is so often performed, we will review it briefly here.

CMA has replaced the classic G-banded chromosome analysis for most indications due to its superiority in detecting small copy number variants, with the ability to detect variants as small as 400 Kb [43]. In contrast, even the highest resolution chromosome analysis is limited to detecting abnormalities of 3.5 Mb or larger [42,43]. While CMA is an excellent first tier test for an individual with nonspecific features, it will not detect single base pair mutations such as those that typically cause SS [43]. Therefore, further testing with DNA sequencing will be required to confirm the diagnosis of SS.

### 3.2. Gene Testing

Many different approaches may be used when pursuing molecular genetic testing for SS. If family history and clinical findings are suggestive of a specific form of SS, one may consider obtaining single-gene testing. Likewise, if suspicion is high for AR SS, a gene panel analyzing only the genes associated with AR SS could be considered. Previously, gene sequencing relied on Sanger Sequencing [44], which is an accurate but slow and costly method [45,46]. However, advancements in next-generation gene sequencing technology, which utilizes methods that sequence millions of short-fragment DNA strands rapidly, allows for a cost-effective method for analyzing multiple genes at one time [45,46,47]. Thus, one could initiate testing for all genes known to be associated with SS—*COL2A1*, *COL11A1*, *COL11A2*, *COL9A1*, *COL9A2*, and *COL9A3*, rather than choose a sequential approach. This is often preferred because it leads to a more cost-effective and rapid diagnosis. If alternative diagnoses are being considered or if the phenotype is unclear, larger gene panels that include other genes or diagnoses of interest could be considered. Because most diagnoses of Stickler Syndrome are due to small exonic and splice-site DNA variations, molecular analysis is estimated to identify a causative genetic variant in approximately 90% of individuals with clinical features of SS [7,8]. Deep intronic variants may be missed by a gene panel, which typically analyzes exonic regions and flanking splice sites, and currently candidate genes are not included in this first-tier testing approach [48,49]. Further studies may be considered if a standard SS gene panel is negative.

### 3.3. Exome and Genome Sequencing

If first tier testing for SS with CMA and gene testing is negative, broad based next generation sequencing with exome or genome sequencing could be considered; however, because the majority of patients with SS will be diagnosed by targeted gene panel, negative first-tier testing suggests that an individual most likely does not have SS but may have an alternate diagnosis [50,51]. Exome sequencing and genome sequencing both use next generation sequencing technology, with exome sequencing focusing on the protein-coding regions of DNA and genome sequencing analyzing the entirety of the genome [47]. The overall diagnostic yield of both exome and genome sequencing is approximately 25–30%, but may be lower in a patient with SS-like features in which first-tier testing with an SS gene panel is negative [50,51].Individuals must undergo genetic counseling and consent for testing, as exome or genome sequencing may reveal unanticipated diagnoses or secondary findings [52,53,54]. Exome and genome sequencing both have limitations, including incomplete coverage of the genome, limitations in detecting DNA deletions and duplications, and limitations in detecting trinucleotide repeats [51].

### 3.4. Interpreting Genetic Testing Results

Clinicians and patients should be aware of possible result outcomes when ordering genetic testing. The ACMG has published guidelines regarding variant classification, which provides a standardized approach by which laboratories classify genetic variants [55]. These variant guidelines outline a scoring system by which a variant may be classified as Benign, Likely Benign, Variant of Uncertain Significance, Likely Pathogenic, and Pathogenic [55]. Variants classified as Benign and Likely Benign are generally not included in testing reports. Likely Pathogenic and Pathogenic variants are considered “positive” results, which provides a patient with a diagnosis and may be medically actionable [55]. Variants with inconclusive evidence to determine pathogenicity are categorized as Variants of Uncertain Significance (VUS) [55]. A VUS should not be considered a molecular diagnosis and in most cases should not be used to alter medical decision making [55]. In some situations, additional investigations with family testing for variant segregation or functional studies could be considered to help confirm the pathogenicity of a VUS [55]. Individuals who receive negative results should be counseled that a negative result does not exclude the possibility of an alternate or undiagnosed genetic etiology [52].

## 4. Conclusions

Stickler Syndrome is a multisystem collagenopathy with significant genetic and phenotypic heterogeneity. Affected individuals may exhibit ocular abnormalities, hearing loss, craniofacial abnormalities, and skeletal abnormalities. Individuals with STL1 and STL2 particularly are at increased risk of retinal detachment with high rates of recurrence and risk of blindness. Thus, a high index of clinical suspicion for Stickler Syndrome with early diagnosis is paramount to preventing adverse outcomes in these individuals. A number of genetic tests may be obtained during a genetics evaluation for Stickler Syndrome, including CMA, gene panels, or exome/genome sequencing. Clinicians and patients should be aware of possible testing outcomes, and patients should be appropriately counseled before testing is obtained. Genetic testing results may be used to help guide treatment and management, assist in testing other at-risk individuals, and provide the patient with recurrence risk to offspring.

## References

[1] Stickler G.B., Belau P.G., Farrell F.J., Jones J.D., Pugh D.G., Steinberg A.G., Ward L.E. (1965). Hereditary Progressive Arthro-Ophthalmopathy. Mayo Clin. Proc..

[2] Snead M.P., Yates J.R. (1999). Clinical and Molecular genetics of Stickler syndrome. J. Med. Genet..

[3] Snead M.P., McNinch A.M., Poulson A.V., Bearcroft P., Silverman B., Gomersall P., Parfect V., Richards A.J. (2011). Stickler syndrome, ocular-only variants and a key diagnostic role for the ophthalmologist. Eye.

[4] Shapiro M.J., Blair M.P., Solinski M.A., Zhang D.L., Jabbehdari S. (2018). The importance of early diagnosis of Stickler syndrome: Finding opportunities for preventing blindness. Taiwan J. Ophthalmol..

[5] Stickler G.B., Hughes W., Houchin P. (2001). Clinical features of hereditary progressive arthro-ophthalmopathy (Stickler syndrome): A survey. Genet. Med..

[6] Robin N.H., Moran R.T., Ala-Kokko L. Stickler Syndrome. https://www.ncbi.nlm.nih.gov/pubmed/20301479.

[7] Annunen S., Korkko J., Czarny M., Warman M.L., Brunner H.G., Kaariainen H., Mulliken J.B., Tranebjaerg L., Brooks D.G., Cox G.F. (1999). Splicing mutations of 54-bp exons in the COL11A1 gene cause Marshall syndrome, but other mutations cause overlapping Marshall/Stickler phenotypes. Am. J. Hum. Genet..

[8] Acke F.R., Malfait F., Vanakker O.M., Steyaert W., De Leeneer K., Mortier G., Dhooge I., De Paepe A., De Leenheer E.M., Coucke P.J. (2014). Novel pathogenic COL11A1/COL11A2 variants in Stickler syndrome detected by targeted NGS and exome sequencing. Mol. Genet. Metab..

[9] Van Camp G., Snoeckx R.L., Hilgert N., van den Ende J., Fukuoka H., Wagatsuma M., Suzuki H., Smets R.M., Vanhoenacker F., Declau F. (2006). A new autosomal recessive form of Stickler syndrome is caused by a mutation in the COL9A1 gene. Am. J. Hum. Genet..

[10] Baker S., Booth C., Fillman C., Shapiro M., Blair M.P., Hyland J.C., Ala-Kokko L. (2011). A loss of function mutation in the COL9A2 gene causes autosomal recessive Stickler syndrome. Am. J. Med. Genet. A.

[11] Faletra F., D’Adamo A.P., Bruno I., Athanasakis E., Biskup S., Esposito L., Gasparini P. (2014). Autosomal recessive Stickler syndrome due to a loss of function mutation in the COL9A3 gene. Am. J. Med. Genet. A.

[12] Ahmad N.N., Ala-Kokko L., Knowlton R.G., Jimenez S.A., Weaver E.J., Maguire J.I., Tasman W., Prockop D.J. (1991). Stop codon in the procollagen II gene (COL2A1) in a family with the Stickler syndrome (arthro-ophthalmopathy). Proc. Natl. Acad. Sci. USA.

[13] Richards A.J., Yates J.R., Williams R., Payne S.J., Pope F.M., Scott J.D., Snead M.P. (1996). A family with Stickler syndrome type 2 has a mutation in the COL11A1 gene resulting in the substitution of glycine 97 by valine in alpha 1 (XI) collagen. Hum. Mol. Genet..

[14] Sirko-Osadsa D.A., Murray M.A., Scott J.A., Lavery M.A., Warman M.L., Robin N.H. (1998). Stickler syndrome without eye involvement is caused by mutations in COL11A2, the gene encoding the alpha2(XI) chain of type XI collagen. J. Pediatr..

[15] Hoornaert K.P., Vereecke I., Dewinter C., Rosenberg T., Beemer F.A., Leroy J.G., Bendix L., Bjorck E., Bonduelle M., Boute O. (2010). Stickler syndrome caused by COL2A1 mutations: Genotype-phenotype correlation in a series of 100 patients. Eur. J. Hum. Genet..

[16] Coussa R.G., Sears J., Traboulsi E.I. (2019). Stickler syndrome: Exploring prophylaxis for retinal detachment. Curr. Opin. Ophthalmol..

[17] Ang A., Poulson A.V., Goodburn S.F., Richards A.J., Scott J.D., Snead M.P. (2008). Retinal detachment and prophylaxis in type 1 Stickler syndrome. Ophthalmology.

[18] Donoso L.A., Edwards A.O., Frost A.T., Ritter R., Ahmad N., Vrabec T., Rogers J., Meyer D., Parma S. (2003). Clinical variability of Stickler syndrome: Role of exon 2 of the collagen COL2A1 gene. Surv. Ophthalmol..

[19] McAlinden A., Majava M., Bishop P.N., Perveen R., Black G.C., Pierpont M.E., Ala-Kokko L., Mannikko M. (2008). Missense and nonsense mutations in the alternatively-spliced exon 2 of COL2A1 cause the ocular variant of Stickler syndrome. Hum. Mutat..

[20] Richards A.J., Meredith S., Poulson A., Bearcroft P., Crossland G., Baguley D.M., Scott J.D., Snead M.P. (2005). A novel mutation of COL2A1 resulting in dominantly inherited rhegmatogenous retinal detachment. Investig. Ophthalmol. Vis. Sci..

[21] Go S.L., Maugeri A., Mulder J.J., van Driel M.A., Cremers F.P., Hoyng C.B. (2003). Autosomal dominant rhegmatogenous retinal detachment associated with an Arg453Ter mutation in the COL2A1 gene. Investig. Ophthalmol. Vis. Sci..

[22] Richards A.J., McNinch A., Martin H., Oakhill K., Rai H., Waller S., Treacy B., Whittaker J., Meredith S., Poulson A. (2010). Stickler syndrome and the vitreous phenotype: Mutations in COL2A1 and COL11A1. Hum. Mutat..

[23] Hanson-Kahn A., Li B., Cohn D.H., Nickerson D.A., Bamshad M.J., University of Washington Center for Mendelian G., Hudgins L. (2018). Autosomal recessive Stickler syndrome resulting from a COL9A3 mutation. Am. J. Med. Genet. A.

[24] Richards A.J., Fincham G.S., McNinch A., Hill D., Poulson A.V., Castle B., Lees M.M., Moore A.T., Scott J.D., Snead M.P. (2013). Alternative splicing modifies the effect of mutations in COL11A1 and results in recessive type 2 Stickler syndrome with profound hearing loss. J. Med. Genet..

[25] Alzahrani F., Al Hazzaa S.A., Tayeb H., Alkuraya F.S. (2015). LOXL3, encoding lysyl oxidase-like 3, is mutated in a family with autosomal recessive Stickler syndrome. Hum. Genet..

[26] Chan T.K., Alkaabi M.K., ElBarky A.M., El-Hattab A.W. (2019). LOXL3 novel mutation causing a rare form of autosomal recessive Stickler syndrome. Clin. Genet..

[27] Schrauwen I., Sommen M., Claes C., Pinner J., Flaherty M., Collins F., Van Camp G. (2014). Broadening the phenotype of LRP2 mutations: A new mutation in LRP2 causes a predominantly ocular phenotype suggestive of Stickler syndrome. Clin. Genet..

[28] Poulson A.V., Hooymans J.M., Richards A.J., Bearcroft P., Murthy R., Baguley D.M., Scott J.D., Snead M.P. (2004). Clinical features of type 2 Stickler syndrome. J. Med. Genet..

[29] Snead M.P., Payne S.J., Barton D.E., Yates J.R., Al-Imara L., Pope F.M., Scott J.D. (1994). Stickler syndrome: Correlation between vitreoretinal phenotypes and linkage to COL 2A1. Eye.

[30] Scott J.D. (1980). Congenital myopia and retinal detachment. Trans. Ophthalmol. Soc. U. K..

[31] Copikova J., Paderova J., Romankova V., Havlovicova M., Balascakova M., Zelinova M., Vejvalkova S., Simandlova M., Stepankova J., Horinova V. (2020). Expanding the phenotype spectrum associated with pathogenic variants in the COL2A1 and COL11A1 genes. Ann. Hum. Genet..

[32] Seery C.M., Pruett R.C., Liberfarb R.M., Cohen B.Z. (1990). Distinctive cataract in the Stickler syndrome. Am. J. Ophthalmol..

[33] Nielsen C.E. (1981). Stickler’s syndrome. Acta Ophthalmol. (Copenh).

[34] Nixon T.R.W., Alexander P., Richards A., McNinch A., Bearcroft P.W.P., Cobben J., Snead M.P. (2019). Homozygous Type IX collagen variants (COL9A1, COL9A2, and COL9A3) causing recessive Stickler syndrome-Expanding the phenotype. Am. J. Med. Genet. A.

[35] Mossey P.A., Little J., Munger R.G., Dixon M.J., Shaw W.C. (2009). Cleft lip and palate. Lancet.

[36] Acke F.R., Dhooge I.J., Malfait F., De Leenheer E.M. (2012). Hearing impairment in Stickler syndrome: A systematic review. Orphanet J. Rare Dis..

[37] Acke F.R., Swinnen F.K., Malfait F., Dhooge I.J., De Leenheer E.M. (2016). Auditory phenotype in Stickler syndrome: Results of audiometric analysis in 20 patients. Eur. Arch. Otorhinolaryngol..

[38] Szymko-Bennett Y.M., Mastroianni M.A., Shotland L.I., Davis J., Ondrey F.G., Balog J.Z., Rudy S.F., McCullagh L., Levy H.P., Liberfarb R.M. (2001). Auditory dysfunction in Stickler syndrome. Arch. Otolaryngol. Head Neck Surg..

[39] Rose P.S., Levy H.P., Liberfarb R.M., Davis J., Szymko-Bennett Y., Rubin B.I., Tsilou E., Griffith A.J., Francomano C.A. (2005). Stickler syndrome: Clinical characteristics and diagnostic criteria. Am. J. Med. Genet. A.

[40] Rose P.S., Ahn N.U., Levy H.P., Ahn U.M., Davis J., Liberfarb R.M., Nallamshetty L., Sponseller P.D., Francomano C.A. (2001). Thoracolumbar spinal abnormalities in Stickler syndrome. Spine.

[41] ACMG Board of Directors (2015). Clinical utility of genetic and genomic services: A position statement of the American College of Medical Genetics and Genomics. Genet. Med..

[42] Miller D.T., Adam M.P., Aradhya S., Biesecker L.G., Brothman A.R., Carter N.P., Church D.M., Crolla J.A., Eichler E.E., Epstein C.J. (2010). Consensus statement: Chromosomal microarray is a first-tier clinical diagnostic test for individuals with developmental disabilities or congenital anomalies. Am. J. Hum. Genet..

[43] South S.T., Lee C., Lamb A.N., Higgins A.W., Kearney H.M., Working Group for the American College of Medical G., Genomics Laboratory Quality Assurance C. (2013). ACMG Standards and Guidelines for constitutional cytogenomic microarray analysis, including postnatal and prenatal applications: Revision 2013. Genet. Med..

[44] Sanger F., Nicklen S., Coulson A.R. (1977). DNA sequencing with chain-terminating inhibitors. Proc. Natl. Acad. Sci. USA.

[45] Mardis E.R. (2011). A decade’s perspective on DNA sequencing technology. Nature.

[46] Pareek C.S., Smoczynski R., Tretyn A. (2011). Sequencing technologies and genome sequencing. J. Appl. Genet..

[47] Koboldt D.C., Steinberg K.M., Larson D.E., Wilson R.K., Mardis E.R. (2013). The next-generation sequencing revolution and its impact on genomics. Cell.

[48] Richards A.J., McNinch A., Whittaker J., Treacy B., Oakhill K., Poulson A., Snead M.P. (2012). Splicing analysis of unclassified variants in COL2A1 and COL11A1 identifies deep intronic pathogenic mutations. Eur. J. Hum. Genet..

[49] Sun W., Xiao X., Li S., Jia X., Zhang Q. (2020). A novel deep intronic COL2A1 mutation in a family with early-onset high myopia/ocular-only Stickler syndrome. Ophthalmic Physiol. Opt..

[50] Shashi V., McConkie-Rosell A., Rosell B., Schoch K., Vellore K., McDonald M., Jiang Y.H., Xie P., Need A., Goldstein D.B. (2014). The utility of the traditional medical genetics diagnostic evaluation in the context of next-generation sequencing for undiagnosed genetic disorders. Genet. Med..

[51] Biesecker L.G., Biesecker B.B. (2014). An approach to pediatric exome and genome sequencing. Curr. Opin. Pediatr..

[52] ACMG Board of Directors (2013). Points to consider for informed consent for genome/exome sequencing. Genet. Med..

[53] ACMG Board of Directors (2012). Points to consider in the clinical application of genomic sequencing. Genet. Med..

[54] Kalia S.S., Adelman K., Bale S.J., Chung W.K., Eng C., Evans J.P., Herman G.E., Hufnagel S.B., Klein T.E., Korf B.R. (2017). Recommendations for reporting of secondary findings in clinical exome and genome sequencing, 2016 update (ACMG SF v2.0): A policy statement of the American College of Medical Genetics and Genomics. Genet. Med..

[55] Richards S., Aziz N., Bale S., Bick D., Das S., Gastier-Foster J., Grody W.W., Hegde M., Lyon E., Spector E. (2015). Standards and guidelines for the interpretation of sequence variants: A joint consensus recommendation of the American College of Medical Genetics and Genomics and the Association for Molecular Pathology. Genet. Med..

